# Smallpox Eradication in Shiraz during 1926 to 1941

**Published:** 2019-05

**Authors:** Mostafa NADIM, Arman ZARGARAN

**Affiliations:** 1. Department of History, School of Literature & Humanity Science, Shiraz University, Shiraz, Iran; 2. Department of History of Medicine, School of Persian Medicine, Tehran University of Medical Sciences, Tehran, Iran

## Dear Editor-in-Chief

Smallpox was always one of the terrible causes of massacre by its epidemics worldwide (1). Although some local societies like nomads of Baluchistan (southeast of Iran) used natural vaccination like method for Smallpox (2), finding vaccination for eradication smallpox is credited for Edward Jenner in 1978 (3). In Iran, by spreading eradication of Smallpox in the early 20^th^ century AD, there was a national effort to control it (4). Fars Province was one of the regions affected by this disease.

**Fig. 1:**
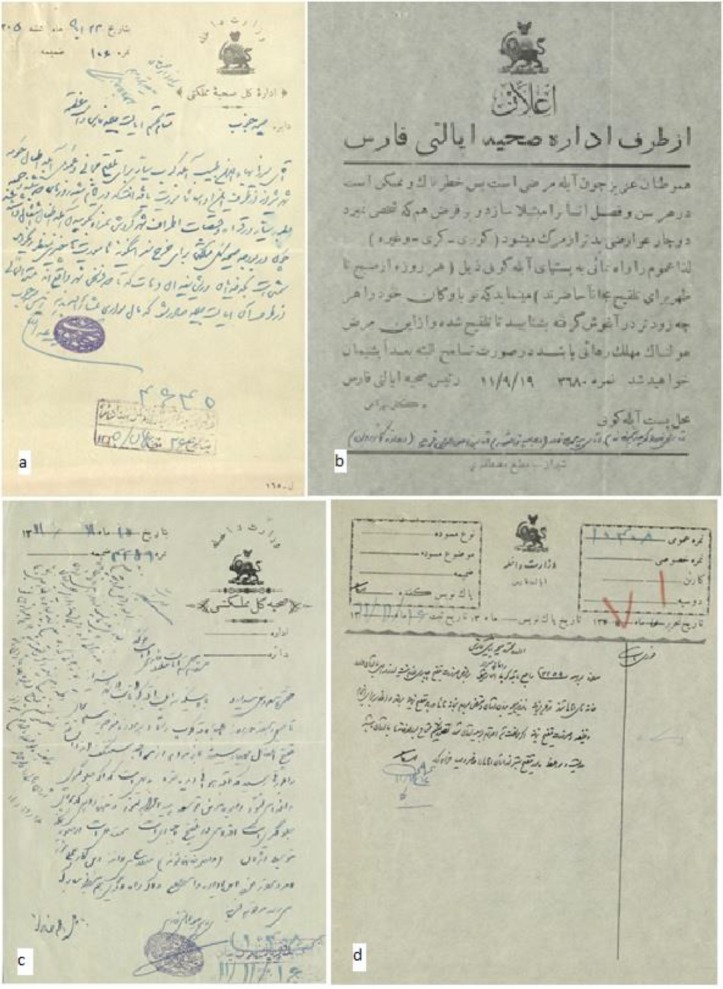
**a)** The letter about appointing; dated 1926; Code no.: 16073; **b)** Public announcement for smallpox vaccination; dated 1932; **c)** The first letter of the dean of health office about smallpox outbreak; dated 1933; **d)** The response of the governor of Fars province; dated 1933; Kept in National Library of Iran, Fars Province branch; from collection no. 14659/293/98

**Fig. 2:**
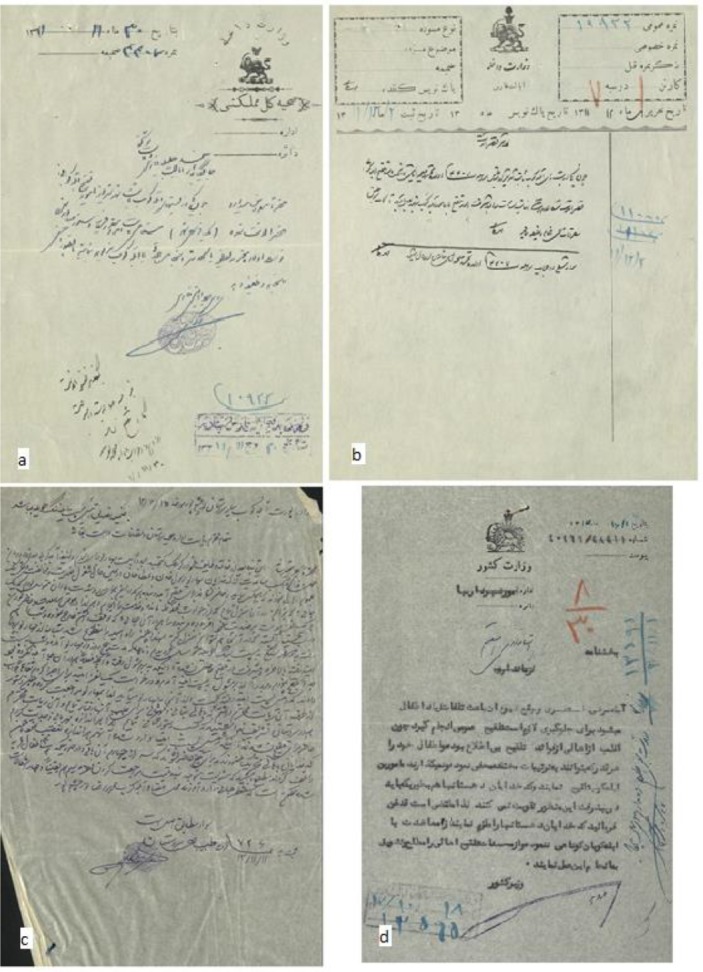
**a)** The letter of the dean of health office asking cooperation of the marshal of *Ghasredasht* village; dated 1933; **b)** The letter of Fars governor order to the marshal of *Ghasredasht* village; dated 1933; **c)** The letter of a vaccination officer to the dean of health office; dated 1934; from collection no. 5830/293/98; **d)** A letter, written by ministry of interior e about the importance of smallpox vaccination; dated 1938; Code no.: 11765; Kept in National Library of Iran, Fars Province branch

There are more than 50 historical documents on smallpox vaccination during first decades of 20^th^ century (first King of Pahlavi dynasty era) kept in Fars Province branch of national library of Iran. The first document dates back to 1926 and the last one belongs to 1945. The type of papers for official ones were white or pale brown papers and simple ones were yellow or green-brown papers like Chinese *Khanbaligh* papers. Documents show that on behalf of government, health office (*Edare-Sehiieh*) directly in Shiraz (center of Fars Province) and also by the help of mobile physicians in towns, informed people the importance of vaccination. One of the oldest documents dates back to 1926 (Fig. 1-a). Based on this letter, *Mirza-Baha al-Din-Tabib* was appointed as vaccination officer (*Abele-Koob*) for the villages around Shiraz. There was not any approved salary for these vaccination officers yet. It was asked from local reeves and doyens to pay them. The second document belongs to Nov 1932 (Fig. 1-b). In this document, Dr. Bahrami, dean of health office in Fars Province, announced people for smallpox dangers and set three locations (*Zanjeerkhaneh* alley, *Darb-Seyed-Zolfaghar*, *Darvazeh* (gate) *Kazeroon*) in Shiraz for smallpox vaccination. It was a public announcement. Less than 2 mounts later, he wrote 2 letters (Fig. 1-c) to governor of the Fars province and declared outbreak of smallpox and people non-cooperation for vaccination. He asked using police force to entry to the people houses and forced vaccination. The governor responded to him (Fig. 1-d) and asked him do not use force though vaccination is mandatory. He also mentioned meet the people with tolerance and only use police force if it would be inevitable. In another letter (Fig. 2-a), dean of health office asked the governor to order to marshal of *Ghasredasht* village to cooperate with the vaccination officer. Following this request, the governor ordered the marshal for cooperation (Fig. 2-b). There was little cooperation with vaccination officers in Shiraz countryside and villages. Doyens and people bothered the officers and prevented vaccination with different methods. One year after the public announcement, a vaccination officer grumbled the marshal and the people of *Sarvestan* (a city in Fars province) for non-cooperation with him. Only 2 months later, this officer wrote another letter (Fig. 2-c) not only grumbling non-cooperation but also asked a police bodyguard for his life protection. He was disappointed. This process was continued for years.

A document, dated 1938 (Fig. 2-d) is a letter written by ministry of interior to governor of Fars Province about the importance of smallpox vaccination and awareness of people and also non-cooperation of doyens of the villages.

The attempt to eradication of smallpox in Iran dates back to Qajar era by the attempts of Prince Abbas Mirza (1789–1833) and then Amir Kabir, prime minister of Iran (1807–1852) (5, 6). Our results showed that complications and difficulties for vaccination in Shiraz were continued after about 100 years starting eradication smallpox in Iran. The peak of these attempts backs to 1931 to 1936, because of the most of documents dated in this period. Although government tried to eradicate smallpox by vaccination, people, doyens, and elders refused accepting it. Even, the governor of the province was not enough aware of the importance of the vaccination and there were some grumblings about him in the historical letters. These complications caused due to cultural poverty in the society at that time. In 40’s decade, these resistances be reduced during increasing people awareness because of decreasing the number of documents and also disease mortality. People finally accepted vaccination and cooperated with government and the attempts were going to be succeeded.
